# Evaluations on the Properties of Polymer and Nanomaterials Modified Bitumen Under Different Aging Conditions

**DOI:** 10.3390/nano15141071

**Published:** 2025-07-10

**Authors:** Shaban Ismael Albrka Ali, Khalifa Salem Gallouz, Ikenna D. Uwanuakwa, Mustafa Alas, Mohd Rosli Mohd Hasan

**Affiliations:** 1 Department of Civil and Construction Engineering, College of Engineering, A’Sharqiyah University, Ibra 400, Oman; 2 Libyan Authority for Scientific Research, Tripoli 00218, Libya; 3 Department of Civil Engineering, Faculty of Engineering, Near East University, 99138 Nicosia, Cyprus; khalifag84@gmail.com (K.S.G.); mustafa.alas@neu.edu.tr (M.A.); 4 Sustainable Asphalt Research Group, School of Civil Engineering, Universiti Sains Malaysia (Engineering Campus), Nibong Tebal 14300, Pulau Pinang, Malaysia; ikeuwanuakwa@gmail.com (I.D.U.); cerosli@usm.my (M.R.M.H.); 5 Gordion Beton, Başkent OSB mah. 16. Cad. No: 25 Malıköy—Sincan/Ankara, 06909 Ankara, Türkiye; 6 School of Civil Engineering, Universiti Sains Malaysia (Engineering Campus), Nibong Tebal 14300, Pulau Pinang, Malaysia

**Keywords:** modified bitumen, polymer, nanomaterials, dynamic shear rheometer, bending beam rheometer, short- and long-term aging

## Abstract

This research evaluates the rheological and mechanical properties of polymer- and nanomaterials-modified bitumen by incorporating nanosilica (NSA), nanoclay (NCY), and Acrylonitrile Styrene Acrylate (ASA) at 5% by weight of the bitumen. The samples were prepared at 165 °C for one hour to obtain homogeneous blends. All samples were subjected to short- and long-term aging to simulate the effects of different operating conditions. The research conducted a series of tests, including consistency, frequency sweep, and multiple creep stress and recovery (MSCR) using the dynamic shear rheometer (DSR) and bending beam rheometer (BBR). The results showed that all modified bitumen outperformed the neat bitumen. The frequency sweep showed a higher complex modulus (G*) and lower phase angle (δ), indicating enhanced viscoelastic properties and, thus, higher resistance to permanent deformation. The BBR test revealed that the bitumen modified with NCY5% has a creep stiffness of 47.13 MPa, a 51.5% improvement compared to the neat bitumen, while the NSA5% has the highest m-value, a 28.5% enhancement compared with the neat bitumen. The MSCR showed that the modified blends have better recovery properties and, therefore, better resistance to permanent deformation under repeated loadings. The aging index demonstrated that the modified bitumen is less vulnerable to aging and maintains their good flexibility and resistance to permanent deformations. Finally, these results showed that adding 5% polymer and nanomaterials improved the bitumen’s’ performance before and after aging by reducing permanent deformation and enhancing crack resistance at low temperatures, thus extending the pavement service life and making them an effective alternative for improving pavement performance in various climatic conditions and under high traffic loads.

## 1. Introduction

Bituminous pavement is widely used in roadway construction due to its water resistance, ease of maintenance, wear resistance, rapid curing time, and overall convenience. It remains the most commonly used paving material worldwide [1,2]. The inherent design of bitumen enables it to withstand bending and stretching under both internal and externa stresses. However, pavements inevitably deteriorate over time due to continuous traffic loading and exposure to environmental conditions [3]. Among these factors, temperature plays a critical role in influencing the performance and durability of bituminous pavements, with significant variations arising from geographical location, diurnal cycles, and seasonal changes [4]. Flexible pavements are especially prone to various types of distress, including cracking, viscoelastic deformation (such as rutting and potholes), and surface inconsistency like raveling and bleeding. Alligator cracking is often associated with sustained heavy traffic loading, whereas transverse and longitudinal cracks are typically caused by thermal shrinkage driven by climate conditions [5]. Neat bitumen, typically used in conventional pavements, exhibits limited resistance to these types of distresses, particularly those induced by thermal fluctuations. Therefore, enhancing pavement performance through incorporation of optimal modifiers, such as polymers and nanomaterials, is crucial, particularly for mitigating thermal cracking.

A widely adopted approach to improving bitumen properties is polymer modification, which involves blending polymers and bitumen at elevated temperatures to produce polymer-modified bitumen (PMB). This technique significantly enhances pavement performance and reduces maintenance requirements. Numerous studies have shown that polymers improve the viscoelastic properties of bitumen, while modifiers like polyethylene and polypropylene enhance its mechanical performance by increasing resistance to various climatic conditions [6,7,8]. For example, Ethyl Vinyl Acetate (EVA) improves penetration, softening point, and ductility, making it more suitable for highway construction. Similarly, Styrene Butadiene Styrene (SBS), an elastomer polymer, significantly enhances viscoelastic behavior and has been shown to improve rutting resistance by 11.8 to 28.4% at elevated temperatures [9]. However, Gaol et al. [10] reported that polymer modification may reduce thermal stability and workability, adversely affecting physical properties like penetration and ductility. In contrast, Akimov et al. (2024) demonstrated that incorporating 8% polymer into bitumen increased the plasticity temperature to 79 °C and elasticity to 82%, thus enhancing overall performance [11]. Bitumen modified with Acrylonitrile Styrene Acrylate (ASA) polymer has shown notable improvements in temperature resistance and permanent deformation, with a reported enhancement of approximately 5%. ASA modification also enhances adhesion and mechanical integrity by altering the bitumen’s internal structure [12]. Aging remains a key factor in determining the long-term performance of polymer-modified bitumen, with the severity and rate of aging depending on both the polymer type and aging conditions.

**Figure 1:**
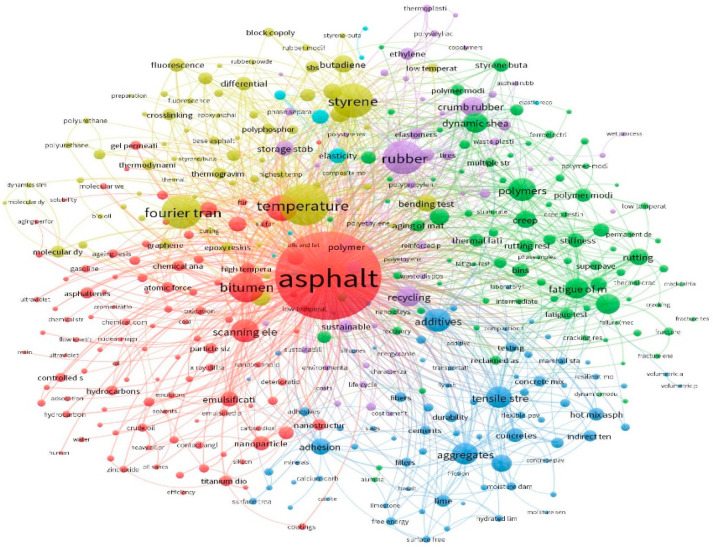
Literature review network visualization.

Beyond polymers, numerous studies have confirmed the effectiveness of nanomaterials in further enhancing the performance of bituminous binders. Nanoparticles significantly improve the aging resistance and durability, primarily by increasing viscosity, which enhances the binder’s resistance to permanent deformation [13,14,15,16]. Nevertheless, practical challenges such as high costs and health risks associated with handling of nanoscale particles [17]. Gholampour et al. [18] conducted a comparative study on the use of nanosilica, titanium dioxide, and calcium carbonate in bitumen. Their findings revealed that nanosilica provided the greatest improvements in physical and rheological properties, while the other nanoparticles yielded limited mechanical benefits. Mashaan [19] similarly demonstrated that nanosilica improved the rutting resistance at elevated temperatures by enhancing the binder’s stiffness and structural cohesion. Nanoclay has emerged as a highly effective additive, improving thermal stability and mechanical strength, while mitigating environmental impacts [20,21]. Figure 1 presents a SCOPUS-based network visualization generated from literature searches using keywords such as “asphalt modification”, “bitumen modification”, “nanomaterial and asphalt”, “nanomaterial and bitumen”, “modified asphalt”, and “modified bitumen”, which returned 5576 relevant studies. The visualization reveals that most of the research predominantly focuses on polymer-based modifiers, such as rubber and styrene, and their influence on the thermal performance of the modified bitumen.

Despite the abundance of research on polymer, nanomaterial, and polymer nanocomposite modified asphalt binders, the aging phenomenon remains insufficiently addressed. Consequently, this study aims to close this gap by systematically analyzing the aging behavior of asphalt binders. The research evaluates physical and rheological properties under unaged, short-term aged, and long-term aged conditions to offer a comprehensive understanding of aging effects and performance implications.

## 2. Materials and Methods

### 2.1. Materials

A Libyan company supplied the 60/70 penetration grade neat bitumen (NB), which is the most commonly used grade for road construction in Libya and was utilized in this study. Three additives were used: nanoclay (montmorillonite (NCY)), nanosilica (NSA), and acrylate–styrene–acrylonitrile (ASA). Each additive was individually blended with the bitumen at a concentration of 5% by the weight of the binder. The concentration was selected based on the previous studies, which identified 5–6% as the optimal modifier content for enhancing bitumen performance [12,22,23,24]. The properties of the bitumen modifiers are summarized in Table 1.

### 2.2. Sample Preparation

**Figure 2:**
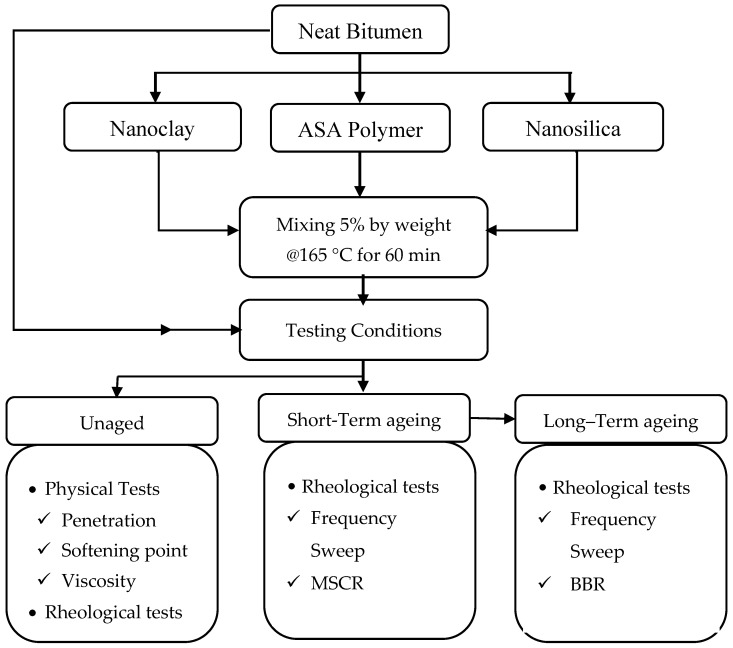
The research flowchart.

The sample preparation began by placing the neat bitumen into a mixing container and heating to 150 °C while mixing at a shear rate of 2000 rpm. After 10 min, the temperature increased to 165 °C, while maintaining a constant shear rate. The 5% additives were then gradually introduced into the bitumen. The mixing process continued for a total duration of 60 min, based on the visual observation of the blend’s homogeneity. Figure 2 presents the flowchart outlining the sequential steps involved in material selection, sample preparation, conditioning protocols, and testing methods employed throughout the experimental program.

### 2.3. Methods and Experimental Procedures

#### 2.3.1. Consistency Test

The bitumen consistency tests were conducted in accordance with their respective ASTM standards. The penetration test, used to assess bitumen hardness, followed the ASTM D5-05 [25] and was performed using a standard needle for five seconds at 25 °C. The softening point test, which evaluates the bitumen’s tendency to flow at elevated temperatures, employed the ring and ball method in accordance with ASTM D36/D36M-09 [26]. To assess the workability of the bitumen suitable temperatures for mixing and construction, the rotational viscosity test was conducted following ASTM D4402-15 [27]. The test utilized a rotational viscometer with 10 g of bitumen and spindle No. 27 rotating at a speed of 20 rpm at two standard test temperatures: 135 °C and 165 °C.

#### 2.3.2. Rheological Test

The study utilized the dynamic shear rheometer (DSR) and bending beam rheometer (BBR) to evaluate the viscoelastic properties of both neat and modified bitumen under various aging conditions. The frequency sweep test was conducted using the DSR on 1 mm thick samples, applying a temperature range from 22 °C + 6 and up to 76 °C and frequency range of 0.1 to 100 rad/s, using a standard 25 mm parallel plate geometry. Additionally, the DSR was employed to perform the Multiple Stress Creep and Recovery (MSCR) test at 64 °C for all samples. The MSCR test included ten cycles at a low stress level (100 Pa) to simulate low traffic volume conditions, followed by ten cycles at a high stress (3200 Pa) to simulate high traffic volume. Each cycle consisted of one second of loading (to induce creep) and nine seconds of recovery, providing insight into the binder’s ability to resist and recover from deformation. This test was performed on samples subjected to short-term aging via the RTFOT procedure. The BBR was conducted to assess the low-temperature performance of the binders, in accordance with ASTM D6648-01 [28]. The test measured creep stiffness and m-value at 0 °C over a 60 s period. According to Superpave specifications, acceptable performance is defined as creep stiffness not exceeding 300 MPa and an m-value of at least 0.300. This test was applied to both short-term and long-term aged samples to evaluate their resistance to thermal cracking.

#### 2.3.3. Aging Conditions

The samples were subjected to both short-term and long-term aging procedures to simulate the oxidative and thermal aging that occurs during mixing, laying, and service life. The short-term aging was performed using the Rolling Thin Film Oven Test (RTFOT), in which 35 g of the bitumen was placed in the RTFOT apparatus and rotated at 15 rpm for 85 min while exposed to an air flow of 4000 ± 200 mL/min at 163 ± 0.5 °C. Following RTFOT, the long-term aging was conducted using the Pressure Ageing Vessel (PAV). The RTFOT-aged samples were subjected to a pressure of 2.1 MPa at 100 °C for 20 h in the PAV chamber. These aging protocols were carried out in accordance with ASTM D2872-19 [29] for RTFOT and ASTM D6521-00 [30] for PAV.

## 3. Results

### 3.1. Consistency Tests

**Figure 3:**
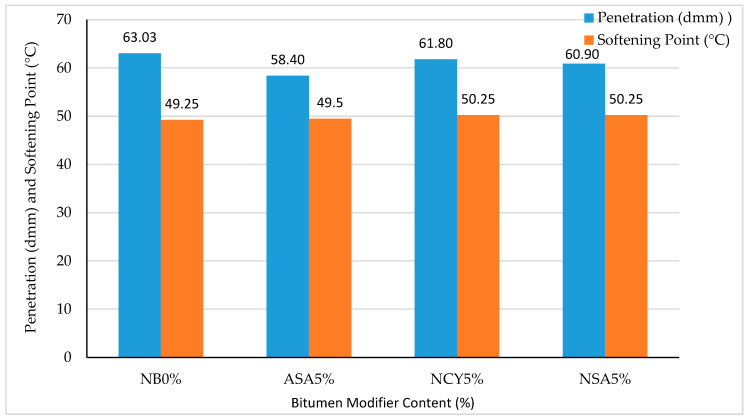
The penetration and softening point of neat and modified bitumen.

Figure 3 shows the results of the penetration and softening point tests conducted on the neat and modified bitumen samples. All binders fall within the standard 60/70 penetration grade specification, confirming their compliance with conventional consistency requirements. The modified samples exhibit lower penetration values compared to the neat binder, reflecting increased hardness and enhanced structural integrity. Among the modified binders, the sample containing ASA5% shows the greatest reduction in penetration (7.34%), followed by NSA5% (3.39%) and NCY5% (1.95%). This reduction in penetration is accompanied by an increase in softening point, indicating a corresponding enhancement in binder stiffness. Specifically, NCY and ASA modifications result in softening point increases of 2.03% and 0.51%, respectively, compared to the neat bitumen. Overall, the modified binders demonstrate slightly altered consistency properties, with increased hardness and thermal resistance that are likely to contribute to improved performance against permanent deformation under service conditions.

**Figure 4:**
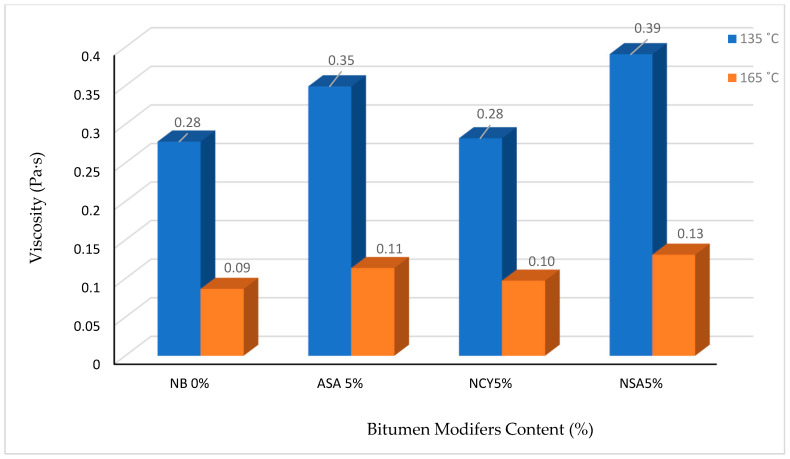
Viscosity of the neat and modified bitumen.

The viscosity of bitumen binders was measured at 135 °C and 165 °C to evaluate the effects of modification with ASA, NSA, and NCY. As shown in Figure 4, the incorporation of these modifiers increases the viscosity of the bitumen, indicating enhanced structural integrity and thermal resistance. At 135 °C, the binder modified with NSA5% exhibits the highest viscosity of (0.391 Pa·s), followed by ASA5% (0.3493 Pa·s) and NCY5% (0.282 Pa·s), while NB0% records the lowest value (0.2777 Pa·s). Importantly, all samples remain within the Superpave specified maximum viscosity limit of less than 3 Pa·s at 135 °C. As expected, viscosity decreases at 165 °C due to temperature-induced softening. The 0%NB shows the most significant reduction of 68.77%, decreasing from 0.2777 Pa·s to 0.0867 Pa·s. In comparison, the modified samples display lower rates of viscosity reduction: 67.45% for ASA5%, 66.51% for NSA5%, and 65.48% for NCY5%. These lower reductions reflect improved thermal stability. Figure 3 and Figure 4 collectively illustrate the penetration and viscosity trends for the modified binders. Notably, NSA5% maintains the highest viscosity across both temperatures, including 0.391 Pa·s at 165 °C, suggesting that it is more viscous among the tested binders. Additionally, its higher softening point supports the conclusion that the nanosilica modification increases the binder’s stiffness and resistance to flow at elevated temperatures.

### 3.2. Frequency Sweep Test

#### 3.2.1. Isochronal Plot

**Figure 5:**
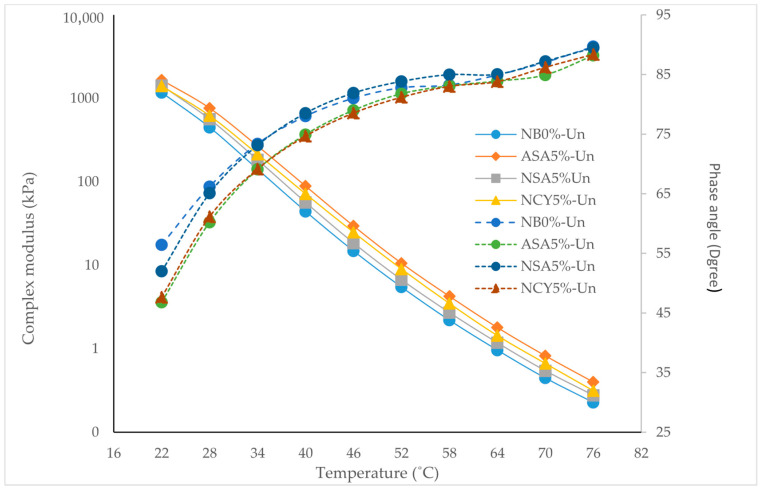
Isochronal plots for the neat and modified bitumen at 10 rad/s.

Figure 5 presents the isochronal plots illustrating the relationship between temperature and the complex modulus (G*) and phase angle (δ) for both neat and modified bitumen samples. As expected for bituminous materials, the complex modulus decreases with increasing temperatures, indicating a reduction in stiffness due to thermal softening. Among the tested binders, the samples modified with NCY5% and ASA5% exhibit superior performance compared to the neat binder, reflecting a significant enhancement in resistance to permanent deformation. The NCY5% binder demonstrates consistently higher complex shear modulus values across the entire temperature range, signifying improved stiffness and load-bearing capacity. Concurrently, all samples show a gradual increase in phase angle with temperature, consistent with a more viscous behavior at elevated temperatures. However, the relatively lower phase angle observed for ASA5% and NSA5% suggest better elastic recovery and enhanced viscoelastic balance. The neat binder (NB0%) exhibits the highest phase angles and the lowest complex modulus, indicating poor resistance to flow and deformation. In contrast, the combination of high modulus and low phase angle observed in NCY5% confirms its superior rheological performance and ability to maintain structural integrity under elevated temperatures. This performance is likely influenced by the structural characteristics of the NCY modifier, which appear to be less dependent on molecular weight, as supported by findings from previous research [31,32].

#### 3.2.2. Master Curve

**Figure 6:**
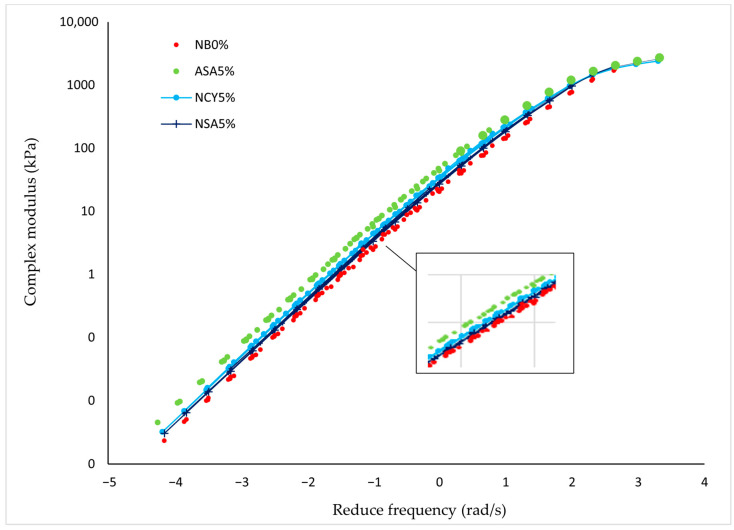
The complex modulus master curves for the neat bitumen and modified samples.

A master curve is used to evaluate the rheological properties of bitumen under various loading conditions using a DSR device. The curve is constructed by selecting a reference temperature and horizontally shifting data collected at other temperatures to create a single, continuous curve, based on the time-temperature superposition. In this study, the numerical method is adopted with a reference temperature of 34 °C. In Figure 6, the *X*-axis represents the reduced frequency (rad/s), which accounts for the temperature-dependent nature of frequency based on the time-temperature superposition equation. The *Y*-axis displays the complex modulus (Pa), representing the material’s resistance to deformation under various loading conditions. The results show that the bitumen modified with ASA5% exhibits a consistently high complex modulus across a wide frequency range, indicating superior resistance to permanent deformation. While the NSA5% and NCY5% modified samples show moderate performance, all modified binders outperform the neat bitumen, highlighting the positive impact of polymeric and nanomaterials additives on the rheological behavior of bitumen. The improved rheological performance is attributed to the ability of the modifiers to interact effectively with the bitumen matrix, forming strong structural bonds. The greater the interaction and dispersion efficiency of the modifier within the binder the better mechanical performance of the modified material [33]. This enhancement is primarily due to the development of a rigid and well-dispersed nanostructured network within the bitumen even at lower concentration [34]. Nanomaterials such as NSA and NCY can improve rheological properties by (1) increasing interfacial adhesion with bitumen components (asphaltenes and maltenes) and (2) reinforcing the binder through both physical and chemical interactions [35].

In summary, the superiority of the ASA polymer lies in its ability to enhance the internal structure of the bitumen, thereby improving its overall performance.

**Figure 7:**
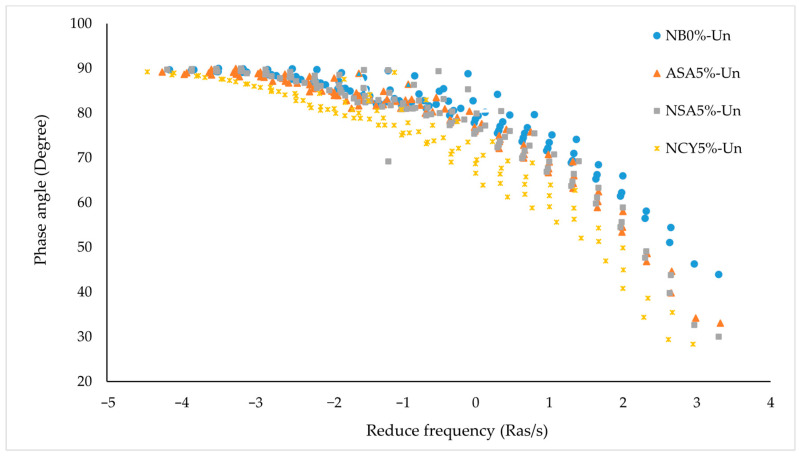
The phase angle master curves for the neat bitumen and modified samples.

Figure 7 presents the master curve of phase angle versus reduced frequency for both neat and modified bitumen samples. The analysis reveals that the inclusion of modifiers significantly influences the rheological behavior of the binders. The neat bitumen (NB0%) exhibits the highest phase angle across most of the frequency spectrum, indicating a predominantly viscous behavior. This suggests limited elastic recovery and reduced resistance to permanent deformation, particularly at lower frequencies, which simulate elevated temperatures or long-term loading conditions. In contrast, the bitumen modified with ASA5% displays a markedly lower phase angle, reflecting a more elastic response and partial preservation of the solid-like characteristics. This shift enhances the binder’s resistance to deformation and offers a favorable balance between viscous and elastic properties. The NAS5% sample exhibits a lower average phase angle compared to NB0%, but higher than ASA5% at certain frequencies, indicating moderate enhancement in viscoelastic behavior. Similarly, the NCY5% binder demonstrates a phase angle slightly lower than that of NB0% and comparable to NSA5%, suggesting modest performance improvement. Overall, the phase angle analysis indicates that ASA5% is the most effective in improving the viscoelastic properties of bitumen, followed by NSA5% and NCY5%. In contrast, NB0% shows the least resistance to permanent deformation under varying service conditions.

#### 3.2.3. Superpave Rutting Parameter

**Figure 8:**
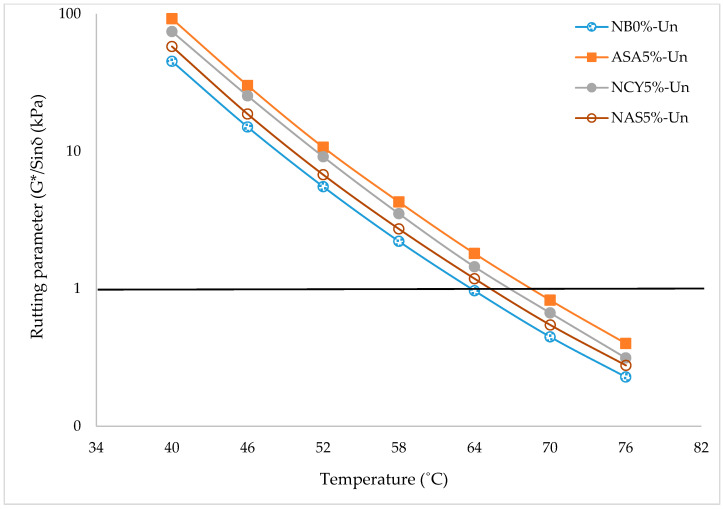
The rutting parameter of the unaged samples.

The rutting parameter (G*/sin (δ)) is one of the Superpave criteria used to assess bitumen’s rutting resistance (permanent deformation) under heavy loads. The G* represents the complex modulus, indicating the material’s overall stiffness, while δ is the phase angle, which describes the ratio of viscous to elastic behavior. Higher values G*/sin (δ) correspond to greater resistance to permanent deformation. As temperature increases, G*/sin (δ) typically decreases due to the softening of the binder. Figure 8 illustrates the Superpave rutting parameters for the NB0% and bitumen samples modified with ASA5%, NSA5%, and NCY5% across a range of temperature (46–76 °C). All modified binders exhibit G*/sin (δ) values exceeding the Superpave minimum threshold of 1.00 kPa for unaged samples at temperatures below 64 °C. Among them, the binder modified with ASA5% demonstrates the best performance, consistently achieving the highest G*/sin (δ) values across all test temperatures. This is followed by NCY5% and NSA5%, while the NB0% shows the lowest resistance to rutting, as expected. A comparison of the percentage improvements in G*/sin (δ) relative to the neat bitumen shows that ASA5% achieves an enhancement of approximately 60%, followed by NCY5% with a 47.5% increase and NSA5% with a 40% increase. These findings confirm the effectiveness of the modifiers, particularly ASA, in significantly improving the rutting resistance of bitumen at elevated service temperatures, thereby making them suitable for use in high-temperature and high-load pavement conditions.

### 3.3. MSCR Test

**Figure 9:**
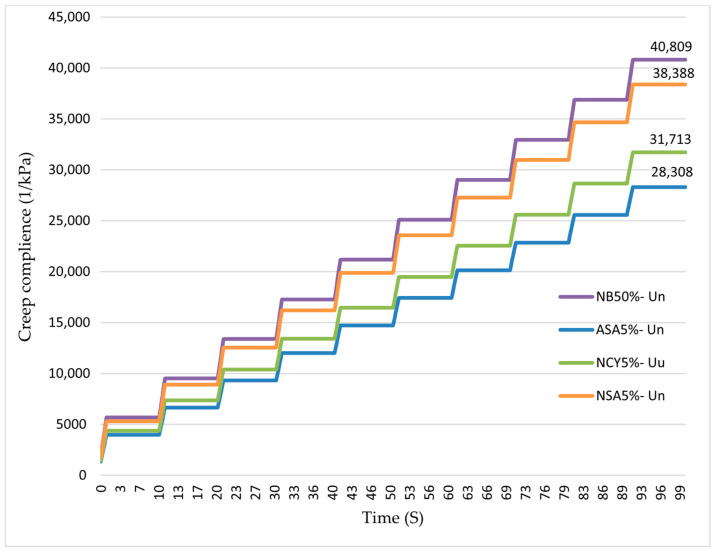
The accumulated creep compliance at 3200 Pa under unaged.

Figure 9 presents the typical strain outputs obtained from the MSCR test assessment of both neat and modified binders, illustrating the relationship between the cumulative time (s) and cumulative deformation. The NB0% exhibits the highest level of deformation throughout the testing period, indicating poor resistance to permanent deformation under repeated loading conditions. In contrast, the modified binders, especially ASA5%, demonstrate significantly reduced cumulative deformation, reflecting enhanced performance under cyclic loading. Specifically, the incorporation of ASA5%, NSA5%, and NCY5% resulted in approximate improvement of 15%, 10%, and 8%, respectively, in reducing cumulative deformation. These findings confirm the effectiveness of both polymeric and nanomaterials modifiers in enhancing the mechanical performance of bitumen. The improved resistance to permanent deformation indicates better long-term pavement performance and durability when subjected to repeated traffic loads.

### 3.4. The Effects of Aging on Modified Bitumen

Bitumen exposed to the environment and temperature undergoes significant changes in its physical and rheological properties that cause aging. The changes during mixing (short-term aging) and the pavement’s service life increase the bitumen’s stiffness and reduce its elasticity, thus increasing the possibility of cracking and diminishing its performance. This section investigates the effects of aging on the performance of the modified bitumen by conducting RTFO and PAV simulations. It is essential to understand the impact of aging to predict bitumen’s long-term performance and select the appropriate additives that ensure optimal durability under the target climatic conditions and repeated traffic load applications.

#### 3.4.1. Effects of Ageing on Isochronal Plots

**Figure 10:**
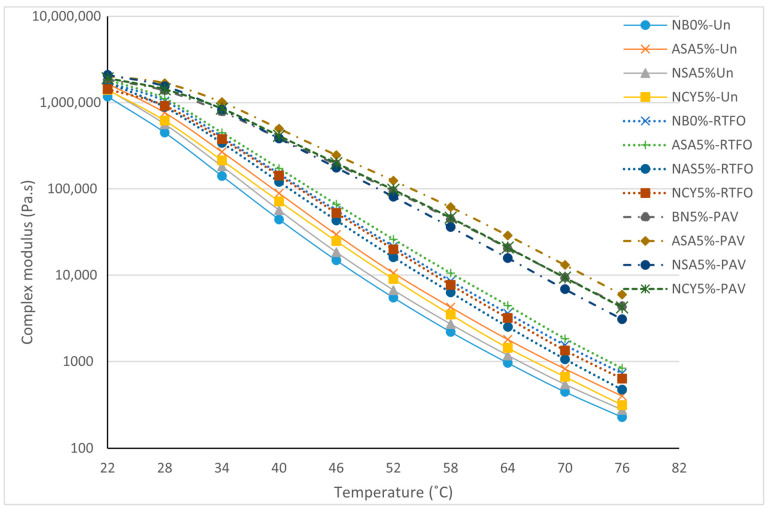
The isochronal plots of G* for the neat and modified samples under aging conditions.

**Figure 11:**
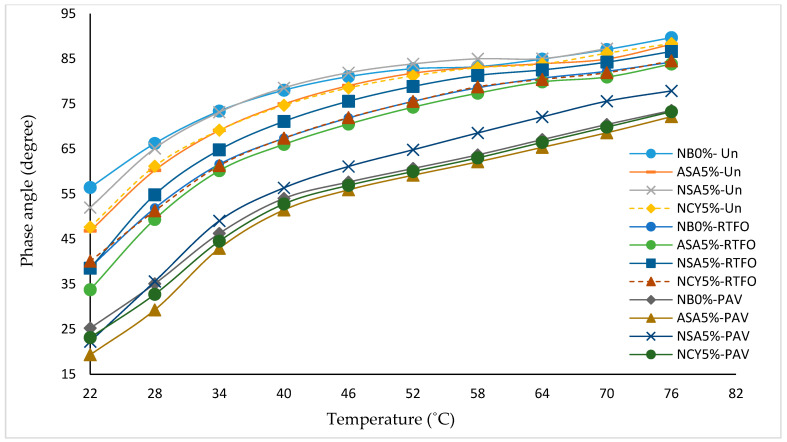
The isochronal plots of δ for the neat and modified samples under aging conditions.

The isochronal plots provide analyses of the rheological properties of the bitumen samples at a constant frequency, offering insight into how temperature influences these properties following aging conditions. As shown in Figure 10, RTFO aging increases the complex modulus of all samples, indicating the loss of volatile components and increased stiffness. A further rise in stiffness is observed after PAV conditioning, with ASA5% exhibiting the highest percentage increase in complex modulus. Interestingly, the nanomaterial modified samples (NSA5% and NCY5%) displayed lower stiffness compared to the neat binder after aging. This behavior indicates their ability to form energy barriers, attributed to their high surface area-to-volume ratio, which promotes both physical and chemical adsorption of polar, oxidizable compounds. This interaction reduces the rate of oxidative reactions, thereby enhancing aging resistance [36,37]. Figure 11 illustrates the changes in phase angle with temperature. As expected, phase angles increase at higher temperatures, reflecting a transition toward more viscous behavior, especially after PAV conditioning. However, all aged samples exhibit overall lower phase angles, indicating an increase in elastic behavior. Among the modified samples, NSA5% and NCY5% demonstrate a lower phase angle than NB0%, signifying improved elasticity and better performance under ageing conditions.

A notable variation in rheological behavior was observed among the different additive dosages under unaged, RTFO, and PAV conditions. Figure 10 illustrates the temperature-dependent behavior of the binders, showing that ASA5% softens more rapidly than NCY5%, likely due to its thermoplastic nature. In contrast, Figure 6, which presents the frequency sweep results at constant temperature, reveals that ASA5% exhibits superior stiffness at higher loading frequencies. These findings indicate that NCY-modified binders perform more effectively at elevated temperatures, while ASA-modified binders demonstrate enhanced stiffness and elasticity at intermediate frequencies, attributable to their elastomeric characteristics.

#### 3.4.2. The Effects of Aging on Master Curve

**Figure 12:**
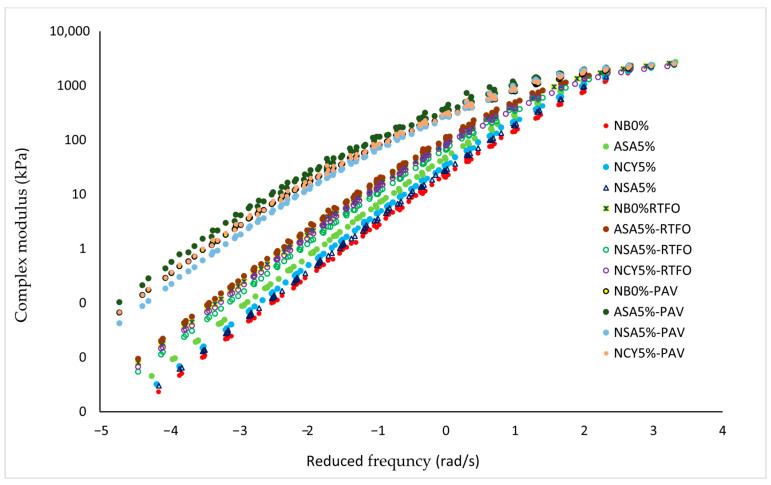
The master curves of the complex modulus for the unaged, RTFO, and PAV samples.

**Figure 13:**
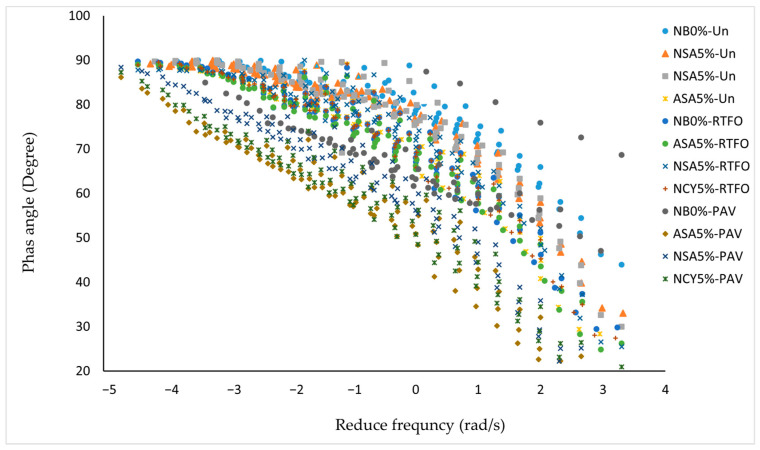
The master curves of the phase angle for the unaged, RTFO, and PAV samples.

Figure 12 and Figure 13 present the master curves for the rheological performance of the complex modulus and phase angle for both modified and neat bitumen samples under various aging conditions (unaged, RTFO, and PAV). The results indicate a slight increase in the complex modulus following RTFO and a more substantial increase after PAV, reflecting the progressive stiffening of the binders. The samples modified with ASA5%, NSA5%, and NCY5% exhibit higher G* values and lower δ compared to the NB0%, indicating improved resistance to deformation and viscoelastic behavior. The reduction in phase angle indicates increased stiffness and more elastic balance, particularly the ASA5% sample, which demonstrates the most favorable rheological properties among the tested blends. The observed gap between the unaged, RTFO, and PAV conditions illustrates the evolution of binder behavior with aging. However, since there is no standardized limit exist for interpreting this gap, it becomes necessary to calculate the aging index (AI) to quantitatively assess the impact of aging on the rheological performance of the modified binders.

**Figure 14:**
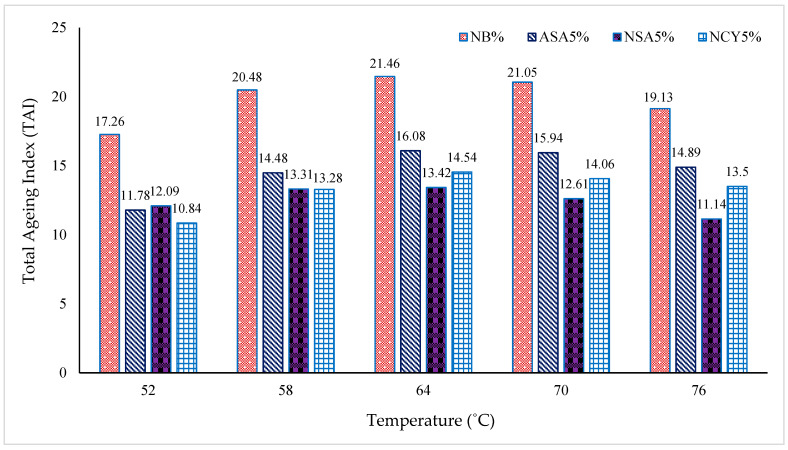
Total Aging Index of the complex modulus at varying temperatures.

The Total Aging Index (TAI) is a critical parameter for evaluating the effects of oxidative and thermal aging on bitumen over time. It quantifies the extent of property changes in the binder due to aging, where higher TAI values indicate a greater susceptibility to aging. As illustrated in Figure 14, the NB0% exhibits the higher TAI values across all tested temperatures, with a peak value of 21.46 at 64 °C. This temperature can be identified as the critical aging point for the binder, reflecting its maximum vulnerability to oxidative degradation, and it is consistent with literature which has shown that chemical oxidation in bitumen does not scale linearly with temperature [38,39]. In comparison, the binder modified with ASA5% shows a moderate improvement, with a gradual reduction in AI values across the temperatures range. With a reduced TAI value of 16.08, its critical aging temperature also occurs at 64 °C, which is the threshold temperature above which the oxidative aging of bitumen significantly accelerates, leading to notable changes in its rheological and chemical properties. These findings demonstrate that bitumen with higher viscosity exhibits improved resistance to aging. The NSA5% binder demonstrates superior aging tolerance, particularly at 76 °C, where it records a significantly lower TAI value of 11.14. Additionally, NCY5%-modified binder shows enhanced aging resistance at lower temperatures, achieving the lowest AI value of 10.84 at 52 °C.

#### 3.4.3. Effects of Aging on Rutting Parameter

**Figure 15:**
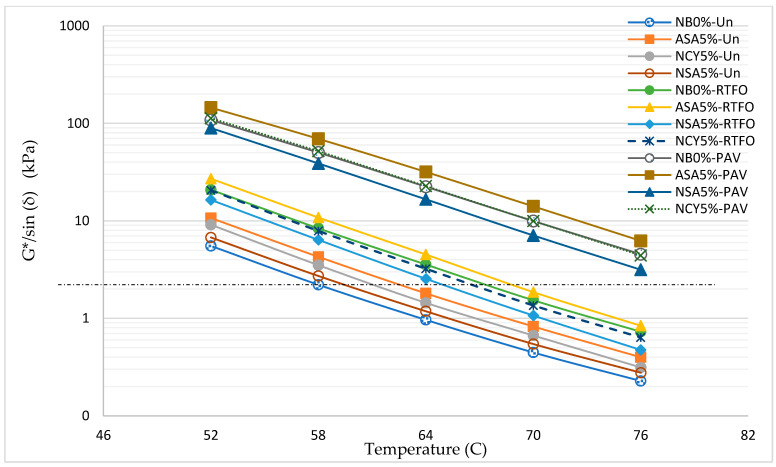
The effects of aging on the rutting parameters of the neat and modified samples.

The rutting parameter G*/sin (δ) is a fundamental Superpave criterion for evaluating the performance of bitumen at elevated temperatures. Figure 15 shows the rutting parameters of tested samples after RTFO and PAV aging. According to the Superpave specifications, the minimum requirement for RTF-aged binders is G*/sin (δ) ≥ 2.2 kPa, while for PAV-aged binders, the maximum limit is G*/sin (δ) ≤ 5000 kPa. As shown in Figure 15, the binder modified with ASA5% outperformed all the other samples, exhibiting the highest rutting resistance across the full temperature range. NSA5% and NCY5% also demonstrate satisfactory performance, particularly between 52 °C and 70 °C, indicating their potential suitability for pavements subjected to high temperatures and traffic loads. In contrast, the NB0% approaches the minimum Superpave limit at elevated temperatures, indicating inadequate rutting resistance under heavy loading conditions. The results for the PAV-aged samples reveal that all binders remain within the acceptable Superpave limit (≤5000 kPa) across the test temperatures. This indicates that all modified and neat binders possess adequate aging resistance and retain essential rheological properties suitable for environments exposed to long-term thermal aging.

**Figure 16:**
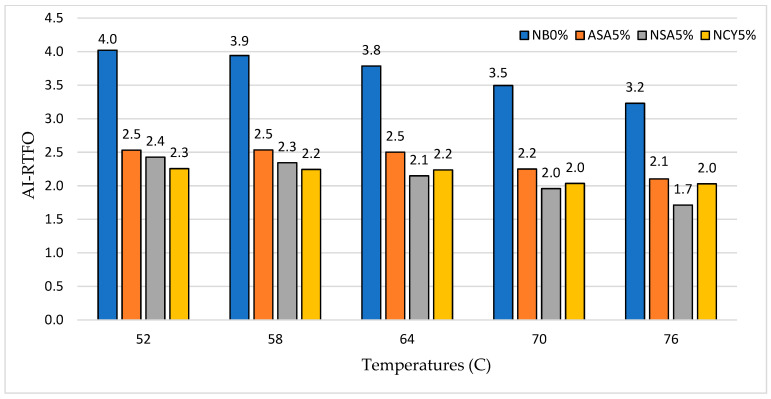
The aging index (RTFO) of the rutting parameter at varying temperatures.

**Figure 17:**
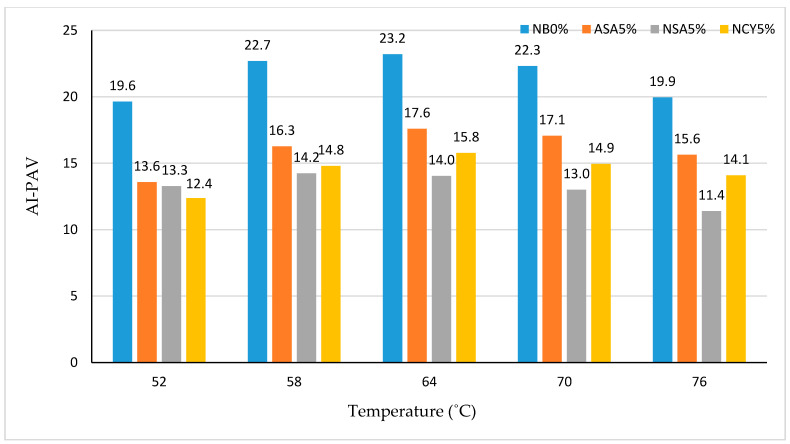
The aging index (PAV) of the rutting parameter at varying temperatures.

Figure 16 and Figure 17 illustrate the AI of the bitumen samples subjected to thermal and oxidative aging. The highest AI is observed for the PAV-aged neat binder (NB0%) at 64 °C, reaching 23.2. This indicates poor aging resistance, as the binder loses a significant portion of its rheological properties over time. In contrast, the modified binders exhibit notably improved aging resistance. Among them, NSA5% shows the greatest reduction in AI up to 42.7% compared to the neat binder (NB0%), followed by NCY5% with a 36% reduction, and ASA5% with 30.6%. Further analysis of the AI values suggests that NSA5% contains the finest particle size among the three modifiers. This supports existing research findings, which indicate the surface area-to-volume ratio of modifiers enhances bitumen’s aging resistance. This enhancement occurs as the reduced molecular mobility and increased adsorption of polar, oxidizable compounds lower matrix entropy and limit the diffusion of oxygen, the primary agent responsible for oxidative ageing [36,37]. Based on these findings, incorporating 5% NSA into the neat bitumen provides optimal aging resistance, particularly for application in high temperatures regions. NSA5% stands out as a cost-effective solution, while NCY5% offers reliable performance across a range of operating conditions.

#### 3.4.4. The Effects of Aging on MSCR

**Figure 18:**
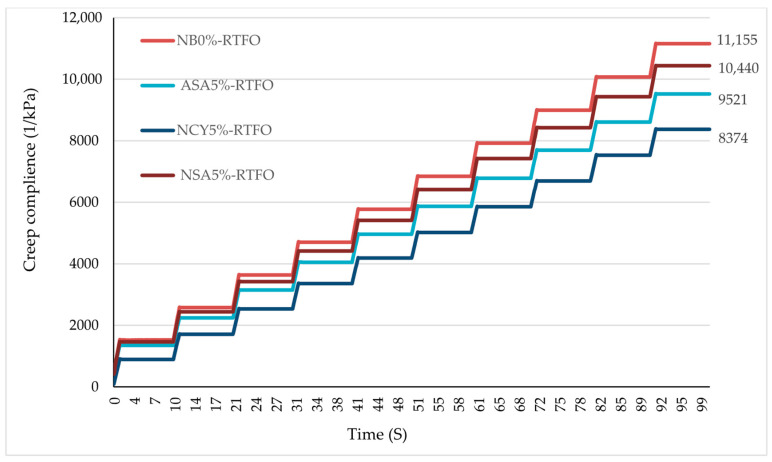
The accumulated creep compliance at 3200 Pa under RTFO.

Permanent deformation (rutting) remains one of the most critical pavement distresses, particularly in hot climates and under high traffic volumes. In this study, all binder samples were subjected to RTFO conditioning, followed by the MSCR test in accordance with AASHTO–T350. The test measured accumulative deformation resulting from repeated loading over a 100 s period. As illustrated in Figure 18, all modified binders significantly outperformed the neat bitumen in terms of rutting resistance. The binder modified with NSA5% exhibited the greatest improvement, achieving a 24.9% reduction in cumulative deformation. This was followed by the binders modified with NCY5% and ASA5%, which showed reductions of 14.6% and 6.4%, respectively, relative to the neat binder. The superior performance of the modified samples, particularly NSA5%, can be attributed to the formation of a strong nanomaterial interaction network within the bitumen matrix. This network acts as a reinforcing filler, promoting polymeric behavior and improving dispersion, which collectively contributes to enhanced resistance to permanent deformation.

**Figure 19:**
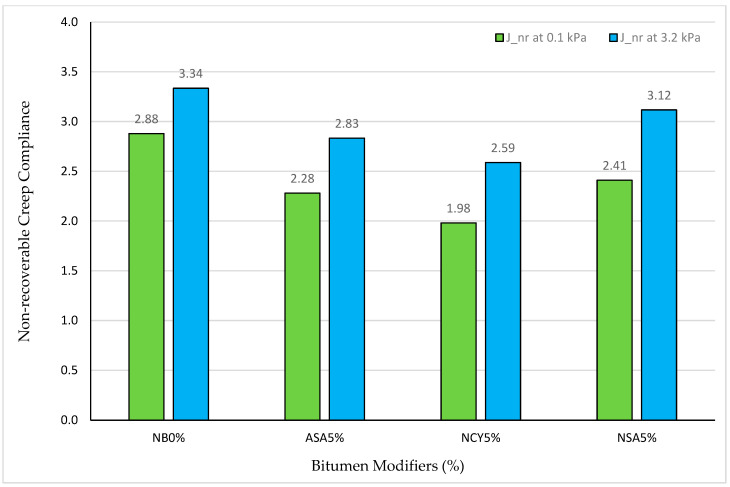
J_nr_ at 0.1 kPa and 3.2 kPa levels of stress.

**Figure 20:**
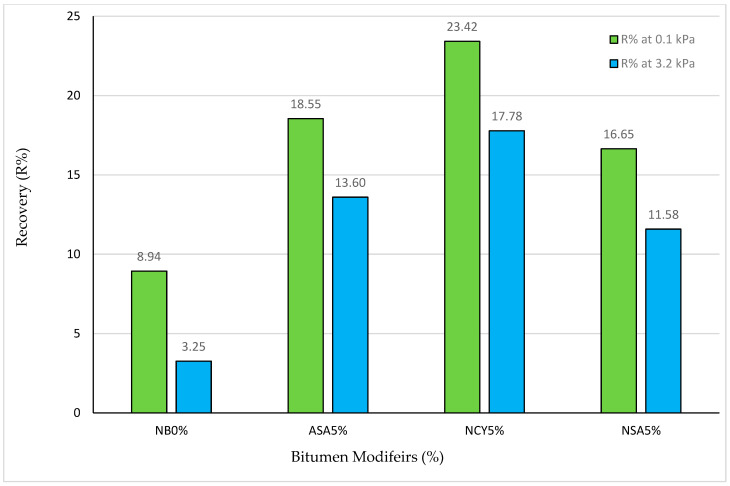
R% at 100 Pa and 3200 Pa levels of stress.

The non-recoverable creep compliance (J_nr_) is a key factor used to evaluate the rutting performance of asphalt binders. A lower J_nr_ value indicates improved resistance to rutting, whereas higher values reflect reduced rutting resistance. Moreover, the recovery percentage is a key parameter for evaluating the elastic behavior of asphalt binder under loading conditions. A higher recovery percentage (R%) indicates greater elastic response and lower susceptibility to permanent deformation. The J_nr_ and R% values provide additional evidence supporting the enhanced rutting and overall superior performance of the modified binders relative to the neat bitumen. As shown in Figure 19 and Figure 20, NCY5% exhibits the most favorable performance in terms of the resistance to permanent deformation. The J_nr_ at 0.1 kPa is around 1.98 kPa^−1^, increasing slightly to 2.59 kPa^−1^ at 3.2 kPa, indicating a stable and flexible response under increased loading. Furthermore, NCY5% achieves the highest recovery rates among all binders (23.42) at 0.1 kPa and 17.78% at 3.2 kPa, demonstrating better elastic recovery.

In comparison, ASA5% displays improved performance over the neat binder, with J_nr_ values of 2.28 and 2.83 kPa^−1^ at 0.1 and 3.2 kPa, respectively, and corresponding recovery percentages of 18.55 and 13.60%. Although this indicates enhanced elasticity, the performance declines with increasing stress, suggesting moderate flexibility. NSA5%, on the other hand, shows a relatively limited enhancement. Its J_nr_ values are 2.41 and 3.12 kPa^−1^ at 0.1 and 3.2 kPa, respectively, with recovery percentages of 16.65 and 11.58. These values reflect an average performance and reduced ability to recover under higher stress levels. Conversely, the neat bitumen (NB0%) consistently demonstrates the weakest performance, with the highest J_nr_ values (2.88 and 3.34 kPa^−1^) and the lowest recovery percentages (8.94 and 3.25%) at the two stress levels. These findings confirm its poor elastic recovery and high vulnerability to permanent deformation following ageing.

#### 3.4.5. The Effects of Aging on BBR

**Figure 21:**
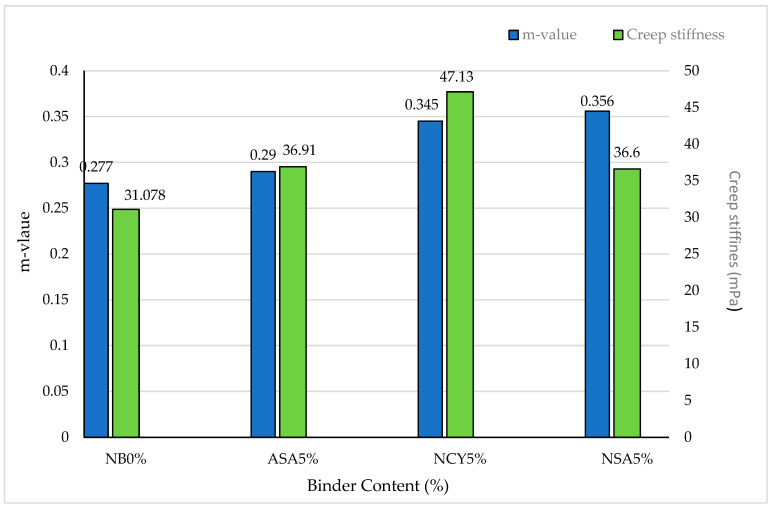
The creep stiffness and m-values of the neat and modified bitumen.

The BBR test was conducted to assess the low-temperatures rheological performance of bitumen at. Figure 21 shows the creep stiffness and m-value measured at 0 °C over a 60 s loading period. The evaluation follows the AASHTO-M320 specifications, which require a creep stiffness (s) of ≤300 MPa and an m-value ≥0.300 to ensure adequate resistance to thermal cracking. The results indicate that the binders modified with NCY5% and NSA5% satisfy both criteria, demonstrating excellent resistance to low-temperature cracking. In contrast, although the binder modified with ASA5% meets the stiffness requirement, it fails to achieve the minimum m-value, indicating a reduced ability to relieve thermal stress. This limitation may compromise its performance in cold climates or environments susceptible to thermal cracking. Similarly, NB0% demonstrates performance comparable to ASA5%, particularly with respect to m-value imitations—an issue commonly associated with neat binders. These findings underscore the importance of bitumen modification in enhancing the low-temperature performance. Specifically, nanomaterial-based modifiers such as NCY and NSA contribute to improved viscoelastic balance, enabling the binder to better withstand thermal stresses and reduce the risk of cracking under cold-weather conditions.

## 4. Conclusions

This study evaluated the performance and properties of neat bitumen (0%NB) and bitumen modified with three additives (ASA, NSA, and NCY) under various aging conditions, using a suite of physical, DSR, and BBR tests. Results indicated that the neat bitumen exhibited the greatest degradation in rheological performance across temperatures and aging regimes, whereas all modified binders effectively mitigated the aging effects.

Notably, the ASA-modified bitumen (ASA5%) demonstrated the most stable behavior under unaged conditions. Master curves revealed enhanced performance of the modified binders across a wide frequency range, characterized by increased complex modulus and reduced phase angles—evidence of improved viscoelastic properties. Low aging index values after RTFO and PAV conditioning confirmed the efficacy of the modifiers in delaying or mitigating aging effects. Although ASA5% delivered satisfactory aging resistance, NSA5% and NCY5% performed even better. Furthermore, all modified binders exceeded the Superpave specification criteria for the rutting resistance: G*/sin (δ) ≥ 2.20 kPa after RTFO and G*/sin (δ) ≤ 5000 kPa after PAV, underscoring their quality and suitability for engineering applications and diverse environments. The findings confirm that both polymeric (ASA) and nanomaterials (NSA and NCY) enhance pavement performance by increasing rutting resistance, reducing aging effects, and improving low-temperature behavior. ASA polymer is particularly well-suited for hot climates, such as tropical regions, while the NSA and NCY nanomaterials are ideal for regions experiencing both high and low temperature extremes.

## Figures and Tables

**Table 1:** Properties of the bitumen modifiers.

Properties	Nanosilica	Nanoclay	ASA
**Formula**	SiO_2_	Montmorillonite type	-
**Color**	White	Gray Brown	White
**Form**	Nano powder	Nano powder	Powder
**Purity**	0.999	0.999	n/a
**Nanoparticle Size (nm)**	30	100	2 × 10^6^
**Molecular Weight (Da)**	6.3–6.49	55	128,000
**Melting Point (°C)**	1600	1300	210–240
**Specific gravity**	n/a	n/a	1.04–1.07

## Data Availability

The original contributions presented in this study are included in the article. Further inquiries can be directed at the corresponding author.

## Abbreviations

The following abbreviations are used in this manuscript:

ASA	Acrylate–Styrene–Acrylonitrile
NB	Neat Bitumen
NSA	Nanosilica
NCY	Nanoclay (Montmorillonite)
DSR	Dynamic Shear Rheometer
BBR	Bending Beam Rheometer
MSCR	Multiple Stress Creep and Recovery
RTFOT	Rolling Thin Film Oven Test
PAV	Pressure Aging Vessel
AI	Aging Index
G*	Complex Shear Modulus
δ	Phase Angle
G*/sin (δ)	Superpave Rutting Parameter
S	Creep Stiffness (in BBR test)
m-value	Slope of creep stiffness curve (BBR)
TSR	Tensile Strength Ratio
SCB	Semi-Circular Bending Test
PMB	Polymer Modified Bitumen
ASTM	American Society for Testing and Materials
AASHTO	American Association of State Highway and Transportation Officials
Pa.s	Pascal-second (unit of dynamic viscosity)

## References

[1] Jin J., Liu S., Gao Y., Liu R., Huang W., Wang L., Xiao T., Lin F., Xu L., Zheng J. (2021). Fabrication of cooling asphalt pavement by novel material and its thermodynamics model. Constr. Build. Mater..

[2] Ignatavicius S., Kavanagh A., Colleran D., Brennan M., Newell S. The use Anionic Bitumen Emulsions in Pavements—A state of the art review. Proceedings of the 7th Eurasphalt and Eurobitume Congress.

[3] ud Din I.M., Mir M.S., Farooq M.A. (2020). Effect of freeze-thaw cycles on the properties of asphalt pavements in cold regions: A review. Transp. Res. Procedia.

[4] Glover C.J., Han R., Jin X., Prapaitrakul N., Cui Y., Rose A., Lawrence J.J., Padigala M., Arambula E., Park E.S. (2014). Evaluation of Binder Aging and Its Influence in Aging of Hot Mix Asphalt Concrete: Technical Report.

[5] Ragnoli A., De Blasiis M.R., Di Benedetto A. (2018). Pavement distress detection methods: A review. Infrastructures.

[6] Kosparmakova S., Seitenova G.Z., Nurakhmetova Z.A., Dyusova R., Jexembayeva A. (2025). A Comprehensive Study on Polymermodifiedbitumen Blends with PP H030 Mixing Parameters and Homogeneity. Kompleks. Ispolz. Miner. Syra=Complex Use Miner. Resour..

[7] Joohari I.B., Giustozzi F. (2022). Waste tyres crumb rubber as a sustainability enhancer for polymer-modified and hybrid polymer-modified bitumen. Int. J. Pavement Eng..

[8] Yang Q., Lin J., Wang X., Wang D., Xie N., Shi X. (2024). A review of polymer-modified asphalt binder: Modification mechanisms and mechanical properties. Clean. Mater..

[9] Praveen Kumar P., KIRAN KUMAR B., Manjunatha S., Gnanamurthy P. (2024). An experimental investigation on the rutting performance of the polymer modified bituminous (PMB) mixes. Civ. Eng. Archit..

[10] Gaol C., Priyono B., Chalid M., Nugraha A.F. (2023). The effect of multilayer plastic waste addition to polymer modified bitumen characteristics. OISAA J. Indones. Emas..

[11] Akimov A.E., Yadykina V.V., Lebedev M.S., Denisov V.P., Inozemtcev S.S., Inozemtcev A.S., Korshunov A.V., Pilipenko A.S. (2024). Development of an Energy-Efficient Method of Obtaining Polymer-Modified Bitumen with High Operational Characteristics via Polymer–Bitumen Concentrate Application. J. Compos. Sci..

[12] Ali S.I.A., Ismail A., Yusoff N.I.M., Karim M.R., Al-Mansob R.A., Alhamali D.I. (2015). Physical and rheological properties of acrylate–styrene–acrylonitrile modified asphalt cement. Constr. Build. Mater..

[13] Tlegenov R., Konkanov M., Jexembayeva A., Korniejenko K. (2024). Application of micro and nano modifying additives in road construction materials. Technobius.

[14] Zhou S., Yan J., Shi B., Li S., Ai C. (2024). Cost-effective enhancement of high viscosity modified bitumen anti-aging properties using organic layered double hydroxide/fume silica nanoparticles composite nanomaterials. J. Clean. Prod..

[15] Alam M.R., Safiuddin M., Collins C.M., Hossain K., Bazan C. (2024). Innovative use of nanomaterials for improving performance of asphalt binder and asphaltic concrete: A state-of-the-art review. Int. J. Pavement Eng..

[16] Iftikhar S., Shah P.M., Mir M.S. (2023). Potential application of various nanomaterials on the performance of asphalt binders and mixtures: A comprehensive review. Int. J. Pavement Res. Technol..

[17] Huang H., Wang Y., Wu X., Zhang J., Huang X. (2024). Nanomaterials for modified asphalt and their effects on viscosity characteristics: A comprehensive review. Nanomaterials.

[18] Gholampour M., Nazari H., Naderi K., Nejad F.M. (2022). Complex reinforcement potential of inorganic nanoparticles in bitumen. Proc. Inst. Civ. Eng.-Constr. Mater..

[19] Mashaan N. (2022). Rutting Performance of Nano-Silica Modified C320 Bitumen. Eng.

[20] Josphineleela R., Diwakar G., Senthilnathan T., Singh H.S., Kumar K.S., Anusuya M. Experimental study on the effects of modification with nanoclay on the properties of an SMA mixture. Mater. Today Proc..

[21] Usman M.I., Murana A.A., Kaura J.M., Ochepo J., Otuoze S.H. (2023). Properties of Bitumen Modified with Nanoclay/Pet (Polyethylene Terephthalate) Blend. Saudi. J. Civ. Eng..

[22] Mubaraki M., Ali S.I.A., Ismail A., Yusoff N.I.M. (2016). Rheological evaluation of asphalt cements modified with ASA polymer and Al2O3 nanoparticles. Procedia Eng..

[23] Saltan M., Terzi S., Karahancer S. (2018). Performance analysis of nano modified bitumen and hot mix asphalt. Constr. Build. Mater..

[24] Shi X., Cai L., Xu W., Fan J., Wang X. (2018). Effects of nano-silica and rock asphalt on rheological properties of modified bitumen. Constr. Build. Mater..

[25] (2017). Standard Test Method for Penetration of Bituminous Materials-American Standard Testing Material.

[26] (2012). Standard Test Method for Softening Point of Bitumen (Ring-and-Ball Apparatus).

[27] (2022). Standard Test Method for Viscosity Determination of Asphalt at Elevated Temperatures Using a Rotational Viscometer.

[28] (2010). Standard Test Method for Determining the Flexural Creep Stiffness of Asphalt Binder Using the Bending Beam Rheometer (BBR).

[29] (2021). Standard Test Method for Effect of Heat and Air on a Moving Film of Asphalt (Rolling Thin-Film Oven Test).

[30] (2000). Standard Practice for Accelerated Aging of Asphalt Binder Using a Pressurized Aging Vessel (PAV).

[31] Uwanuakwa I.D., Adamu M., Ali S.I.A., Akpinar P., Hasan M.R.M., Shariff K.A., Umar I.K., Haruna S. (2023). Effect of polymer molecular weight on the rheology of SBS polymer-modified asphalt binder. Innov. Infrastruct. Solut..

[32] Schaur A., Unterberger S.H., Lackner R. (2021). Impact of molecular structure of PP on thermo-rheological properties of polymer-modified bitumen. Constr. Build. Mater..

[33] Filonzi A., Sabaraya I.V., Hajj R., Das D., Saleh N.B., Bhasin A. (2018). Evaluating the Use of Nanomaterials to Enhance Properties of Asphalt Binders and Mixtures.

[34] Santoro L. (2018). Effects of Nano-Sized Additives on Bituminous Binders: Rheological and Morphological Characterization. Doctoral Dissertation.

[35] Broering W.B., de Melo J.V.S., Manfro A.L. (2022). Incorporation of nanoalumina into a polymeric asphalt matrix: Reinforcement of the nanostructure, improvement of phase stability, and amplification of rheological parameters. Constr. Build. Mater..

[36] Crucho J., Picado-Santos L., Neves J., Capitão S. (2019). A review of nanomaterials’ effect on mechanical performance and aging of asphalt mixtures. Appl. Sci..

[37] Omar H.A., Katman H.Y., Bilema M., Ahmed M.K.A., Milad A., Md Yusoff N.I. (2021). The effect of ageing on chemical and strength characteristics of nanoclay-modified bitumen and asphalt mixture. Appl. Sci..

[38] Cao W., Fini E. (2022). A molecular dynamics approach to the impacts of oxidative aging on the engineering characteristics of asphalt. Polymers.

[39] Lu X., Isacsson U. (2000). Artificial aging of polymer modified bitumens. J. Appl. Polym. Sci..

